# Factors influencing vaccine hesitancy toward non-covid vaccines in South Asia: a systematic review

**DOI:** 10.1186/s12889-025-22462-4

**Published:** 2025-04-02

**Authors:** Sophie C.W. Stuetzle, Matthew Willis, Ewelina Julia Barnowska, Ann-Kristin Bonkass, Anil Fastenau

**Affiliations:** 1 Department of Global Health, Institute of Public Health and Nursing Research, University of Bremen, Bremen, Germany; 2 Marie Adelaide Leprosy Center, Karachi, Pakistan; 3 School of Medicine, Dentistry & Biomedical Sciences, Queen’s University Belfast, Belfast, BT7 1NN UK; 4 evaplan GmbH at the University Hospital Heidelberg, Heidelberg, Germany; 5 German Leprosy and Tuberculosis Relief Association (DAHW), Wuerzburg, Germany

**Keywords:** Vaccine hesitancy, Vaccine, South Asia, Immunisation, Communicable diseases

## Abstract

Declared as one of the ten most pressing threats to global health in 2019, the complexity around vaccine acceptance and hesitancy has once again gained great momentum following the COVID-19 pandemic. Lack of vaccine acceptance may endanger the mission of improving vaccine uptake globally to tackle pandemics, reduce morbidity and mortality of preventable diseases and to prevent antibiotic resistance worldwide. Countries of the global south, including South Asian Association for Regional Cooperation (SAARC) countries are especially affected by the dangers of low vaccination uptake and continue to show decreases in coverage in recent years. This paper examines factors contributing to vaccine hesitancy in south Asia and the extent to which they are context and disease specific, guided by a modified version of the WHO SAGE 5 C model by Razai et al. Three databases were searched for peer-reviewed articles by using a comprehensive search strategy. Results from 44 quantitative, qualitative and mixed-method studies were included in the systematic review and appraised for quality, thematically analyzed and mapped onto the 5 C model. findings from India, Pakistan, Bangladesh, Nepal and Afghanistan showed vaccine hesitancy to be context and vaccine-specific within different settings of the countries. The main factors contributing to vaccine hesitancy can be identified as narrow confidence in vaccines, miscommunication, and lack of knowledge embedded into overarching constraints and contexts. To tackle vaccine hesitancy in SAARC countries, interventions need to be co-created by communities and mutual trust needs to be ensured, for instance through increased equity in knowledge distribution.

**Supplementary Information:**

The online version contains supplementary material available at 10.1186/s12889-025-22462-4.

## Introduction

The impact of vaccination on the health of the world’s peoples is hard to exaggerate [1, 2]. As a key component of global health, immunization is estimated to save five million lives annually, reducing the mortality and morbidity of various diseases [2, 3]. Vaccines contribute to economic and social systemic improvements as well as strengthening health security [4]. Lack of vaccine acceptance may endanger the mission of improving vaccine uptake globally to tackle pandemics, reduce morbidity and mortality of preventable diseases. The resultant increase in vaccine preventable bacterial infections and subsequent antibacterial usage can also lead to an increase in antibiotic resistance [5].

Despite an increase in global vaccination coverage by up to 80% since the 1980’s, United Nations Children’s Fund (UNICEF) reported a decrease in global routine vaccination rates by at least 5% in 2021 [6]. Whereas the decline in vaccination coverage is not evenly distributed across the world, countries in south Asia, have shown a stronger decrease in recent years [7]. The decline is partly attributable to the widening poverty gap between and within countries due to south Asia’s rapid but unequally distributed socioeconomic development [8]. Economic liberalization continues to contribute to social inequalities in the region. While the proportion of the population in Sri Lanka and Bhutan living below the international extreme poverty line (less than 2.15$ a day) is below 3%, Pakistan and The Maldives range from seven to eight% of the population, followed by fourteen and nineteen% of the population of Bangladesh and Nepal respectively [9, 10]. In the context of vaccine inequity, low Socioeconomic Status (SES) can be affiliated with vaccine underutilization resulting from time and language barriers, restricted accessibility to healthcare, high costs and limited health literacy. This has been described as a link between poverty and lack of vaccination equity [9]. On the one hand vaccines not only tackle diseases but also poverty, as they prevent families from facing catastrophic healthcare expenses [11],. On the other hand, the COVID-19 pandemic has once again surfaced the unequal distribution and uptake of vaccination between “rich” and “poor” countries, where the poorest countries were also the most poorly vaccinated [12]. In light of that, it is crucial to place focus on the eight South Asian Association for Regional Cooperation (SAARC) countries, as they account for some of the most poverty-inflicted places globally with a combined population of more than two billion people [13]. Pakistan and Afghanistan are the only two countries in the world where Poliomyelitis is still endemic, emphasizing the need for high immunization rates in these countries and bordering countries [7].

### Variation in vaccine rates and contributing factors

As previously demonstrated, SAARC countries (Afghanistan, Bangladesh, Bhutan, India, Maldives, Nepal, Pakistan and Sri Lanka) differ greatly in terms of poverty rates. Country-specific vaccine rates vary widely as well. For instance, Bangladesh and Nepal rank highest at Diphtheria-Pertussis-Tetanus (DPT)-vaccination rates with almost 90% coverage, while Afghanistan’s Pakistan’s and India’s immunization rates are the lowest in the SAARC region [14]. Regarding the measles vaccine, only 60% of Pakistani children received the vaccine at all, Afghan children even less, whereas India reports immunization rates of 70% [7]. Further, limited data on the Rotavirus vaccine indicates India to display coverage of under 40%, whereas Afghanistan and Pakistan reach 60% [14]. Overall, data shows influenza vaccine coverage to be low across all south Asian regions [14]. Evidently, SAARC countries strongly demonstrate vaccine rates to be country and vaccine specific [15].

Many countries struggle with deficient infrastructures that in turn hamper the equal supply and accessibility of vaccinations. Thereby, sufficient water supply and sanitation, transport, waste management, and digitalization are essential to ensure sufficient vaccine uptake [16]. A robust health infrastructure is also essential for the effective implementation of vaccination programs. This includes the establishment of vaccination clinics accessible to local populations, an adequate workforce of health professionals, the availability of vaccines and necessary vaccination equipment, and a reliable cold chain system to ensure the proper storage and distribution of vaccines.

Additionally, geopolitical tensions between and within countries, like the Kashmir conflict between Pakistan and India and the separatist movements in Pakistan as well as the ongoing humanitarian crisis in Afghanistan since the Taliban usurped power, impacts vaccine uptake greatly [17, 18]. Outdated data recording and insufficient planning also impact vaccine uptake [19]. For example, the last national census in Pakistan was in 1993, making it difficult to forecast the demand for vaccination or to reach those who are yet to receive specific vaccines [19].

### Vaccine hesitancy

Declared as one of the ten most pressing threats to global health in 2019, the complexity around vaccine acceptance and hesitancy has gained great momentum following the COVID-19 pandemic [20]. Generally, perceptions towards vaccine acceptance are set on a continuum from total acceptance to complete refusal with those hesitant towards vaccines in between the two extremes [15]. Overall, vaccine hesitancy refers to “a delay in acceptance or refusal of vaccines despite the availability of vaccine services” [21]. In the case of COVID-19, the global circulation of misinformation, lack of awareness, the inconsistent framing and blaming of vaccines and their linkage to side effects as well as the short development period and production of vaccines contributed to public mistrust among a significant proportion of the population and as such is a distinct phenomenon [22]. Although research on vaccine hesitancy in south Asia is limited, the recent COVID-19 pandemic displayed the prevalence of COVID-19 vaccine hesitancy [23]. In Bangladesh, for example, 54% of the population expressed doubts about the efficacy of the vaccine, in Pakistan only 4% trusted in western vaccines [24]. Such distrust is specifically rooted in suspicions directed towards Pakistan’s polio-eradication campaigns following political misuse by the CIA [24]. As previously described, vaccine hesitancy is a context and vaccine specific behavioral phenomenon with numerous components [15]. As the world aims to reach universal health coverage by 2050 and to achieving SDG 3 Target 3.b “development assistance and vaccine coverage“ by 2030, it is essential to study underlying factors of vaccine hesitancy [25–27]. The World Health Organization’s (WHO) Strategic Advisory Group of Experts on Immunization (SAGE) Working Group has developed an explanatory framework to identify and address vaccine hesitancy, the SAGE 5 C Model [26].

Using an adapted version of the theoretical model by Razai et al. [28], psychological, contextual, and socio-demographic obstacles to vaccination were classified and pinpointed. Previous literature describes vaccination acceptance as a “behavioral outcome resulting from a complex decision-making process that can be impacted by a wide range of factors” [15]. Such range of factors can be displayed by the five C framework that believes decisions to be shaped by interactions between confidence, complacency, convenience/constraints, communication, and context [27].

This review aims to highlight current knowledge about vaccine hesitancy to toward non-covid vaccines in the South Asian context, and to map this knowledge against a modified version of the 5 C framework [28]. The results will allow for the suggestion of pathways to address this hesitancy for better health promotion and disease prevention. The review will also explore the nature of vaccine hesitancy in relation to different countries, contexts, and vaccines. Moreover, it will investigate the psychological and socio-demographic barriers that impede vaccination uptake.

## Methods

### Search strategy

A systematic review was conducted in accordance with the PRISMA guidelines in the databases *PubMed*,* Embase* and *Web of Science* by three authors MW, SCWS and EJB with the final search taking place on 25/09/2024. The PRISMA checklist for this review is attached as SI 1. This review aims to examine underlying factors contributing to vaccine hesitancy in south Asian countries. Thereby, search terms consisting of controlled vocabulary for Pubmed (MeSH), Embase (Emtree) and free text terms around vaccines, hesitancy, and the setting (and synonyms) were used in the search strategy. The developed search string for the database PubMed was adapted to Embase and Web of Science. The search terms are connected by the Boolean Operators “AND“, “OR“ and “NOT“ and applied in an Abstract and Title search to refine the overall search (for the full search strategy and terms used, see Supplementary File SI 2).

### Eligibility criteria

The systematic review search is restricted to the English and German language, therefore research papers in other languages were excluded from the search. Furthermore, the publication period of assessed papers was not narrowed down to a specific timeline, to consider all factors that shaped vaccine hesitancy throughout history. Only published, peer-reviewed research articles were considered in the search, grey literature was excluded. Qualitative, quantitative and mixed methods studies were included. Along with that, only studies from the SAARC countries were included. The inclusion and exclusion criteria are summarized below in Table 1.

**Table 1:** Inclusion and exclusion criteria

Inclusion	Exclusion
Studies in German and English Language	Studies not in German or English Language
Published, Peer reviewed articles	Non-published, peer-reviewed articles
Relevant data for 5c model	Relevant data for 5c model
Studies from SAARC countries	Studies outside of SAARC
Standard Vaccines	COVID-19 vaccines

### Data collection

The search strand was created by SCWS in consultation with MW and AF and is summarized in SI 2. The final search took place on the 25/09/2024. MW, SCWS and EJB then independently screened titles and abstracts using Rayyan software [29], followed by full-text screening. This was carried out independently by each author, where discrepancies occurred and a consensus was reached after discussion between MW, SCWS and EJB.

### Data extraction and appraisal

Included studies were extracted using a pre-defined extraction sheet in Excel by SCWS and MW. Categories for extraction were study design, sample, type of vaccine, measures, outcome, setting and reasons for vaccine hesitancy (the resulting table of study characteristics can be found in SI 3). Quality appraisal of the selected papers was conducted by SCWS, MW and EJB. Due to the nature of the selected papers, appropriate tools complying with quantitative, qualitative, and mixed-method study designs were applied to evaluate the Risk of Bias. For mixed-method studies the Mixed Method Appraisal Tool (MMAT) [30] was used, for qualitative studies the NICE checklist [31], and for quantitative studies the Critical Appraisal Toolkit (CAT) [32]. Quality appraisal results are summarized in SI 4.

### Data analysis

The collected findings were thematically analyzed [33] and reported narratively. Inductive coding for themes was complemented by deductive coding against the 5 C framework through a thematic analysis to gain a profound understanding of the gathered evidence. Hereby, information was screened for thematic patterns along with analyzing the extent to which the selected papers referred to the 5 C framework and whether other influences on vaccine hesitancy were shown. In general, findings and characteristics of the collected studies were summarized, analyzing and investigating relationships and the heterogeneity between studies [34].

### Theoretical framework

The 5Cs by Razai et al. [28] are divided into confidence, complacency, convenience/constraints, communication, and context. In the 5 C model proposed by SAGE, we observed that the concepts of collective responsibility and calculation overlap with the elements of confidence, convenience, and complacency. To emphasize the significance of communication and context, we chose to include these aspects instead. Additionally, while the original SAGE model touches on awareness through the concept of confidence, we found this coverage to be limited. Therefore, through inductive coding based on collected data, we incorporated awareness and knowledge, as well as reasons to vaccinate, into our revised framework.

C*onfidence* goes along with trusting in the efficacy and safety of specific vaccines and the system that provides them. Therefore, competencies of health systems and its staff are taken into consideration as well as underlying intentions of health professionals and policymakers controlling the distribution of vaccines. Hence, low confidence in vaccines results from a lack of trust in health systems and the government, potentially driven by misinformation and tunneled perceptions towards vaccine-risks [16].Thus, numerous people may perceive the great benefits of vaccines to be outweighed by potential risks and side effects [27]. The second C *complacency* transpires when individuals do not identify vaccines as efficient measures to prevent diseases, instead the risk of vaccine refusal is minimal to them. Therefore, individuals perceive vaccinations as unnecessary. This goes along with the fact that individuals may tend to direct their attention towards other health priorities requiring more notice. Besides, complacency interacts with self- efficacy and the individuals’ greater societal interests in achieving herd immunity [26, 27]. The third C *convenience*, recently changed to *constraints*, takes geopolitical and economic, physical, and structural reasons that underlie individual decision-making into account. *Convenience* is therefore associated with vaccine accessibility, production, infrastructures, costs, supply reliability as well as mobility. Depending on specific circumstances, such factors may act as conveniences, positively impacting people’s vaccination uptake or as constraints, negatively affecting individuals’ ability to receive vaccines without facing numerous hurdles along the way. Therefore, *convenience/constraints* indicates the possibility of vaccine hesitancy to arise unintentionally, but to be shaped by surrounding circumstances in addition to individual psychological elements [26, 27]. The fourth C addresses the influence of *communication* on vaccine acceptance and hesitancy. Previously addressed by the World Health Organization as an “infodemic”, uncertainty towards vaccinations is amplified through (social) media platforms. Thereby, the vast spread of misinformation and framings around certain narratives elevates previous skepticism and anxiety, ultimately encouraging anti-vaccination- conspiracy theories, confusion, and distrust [27]. Finally, the fifth C *context* includes SES, religion, ethnicity, and occupation. Whereas the term vaccine hesitancy implies to be shaped solely by individual health behaviors, overarching components are often overlooked. For instance, structural factors including barriers to access, religion or systemic racism affecting vaccine uptake, magnifying previously existing inequalities in south Asian countries [7]. The lines between the five C’s are easily blurred, as all interact and influence one another during the individual decision-making process. Whereas confidence, complacency and convenience/constraints act as specific psychological determinants, communication and context also identify as tools shaping the first three C’s. Hereby, vaccine hesitancy is placed on a matrix, where factors such as SES and education can influence vaccination behavior into the direction of both acceptance and hesitancy, thus reasons to vaccinate was included as an additional criteria [16].

## Results

**Fig. 1:**
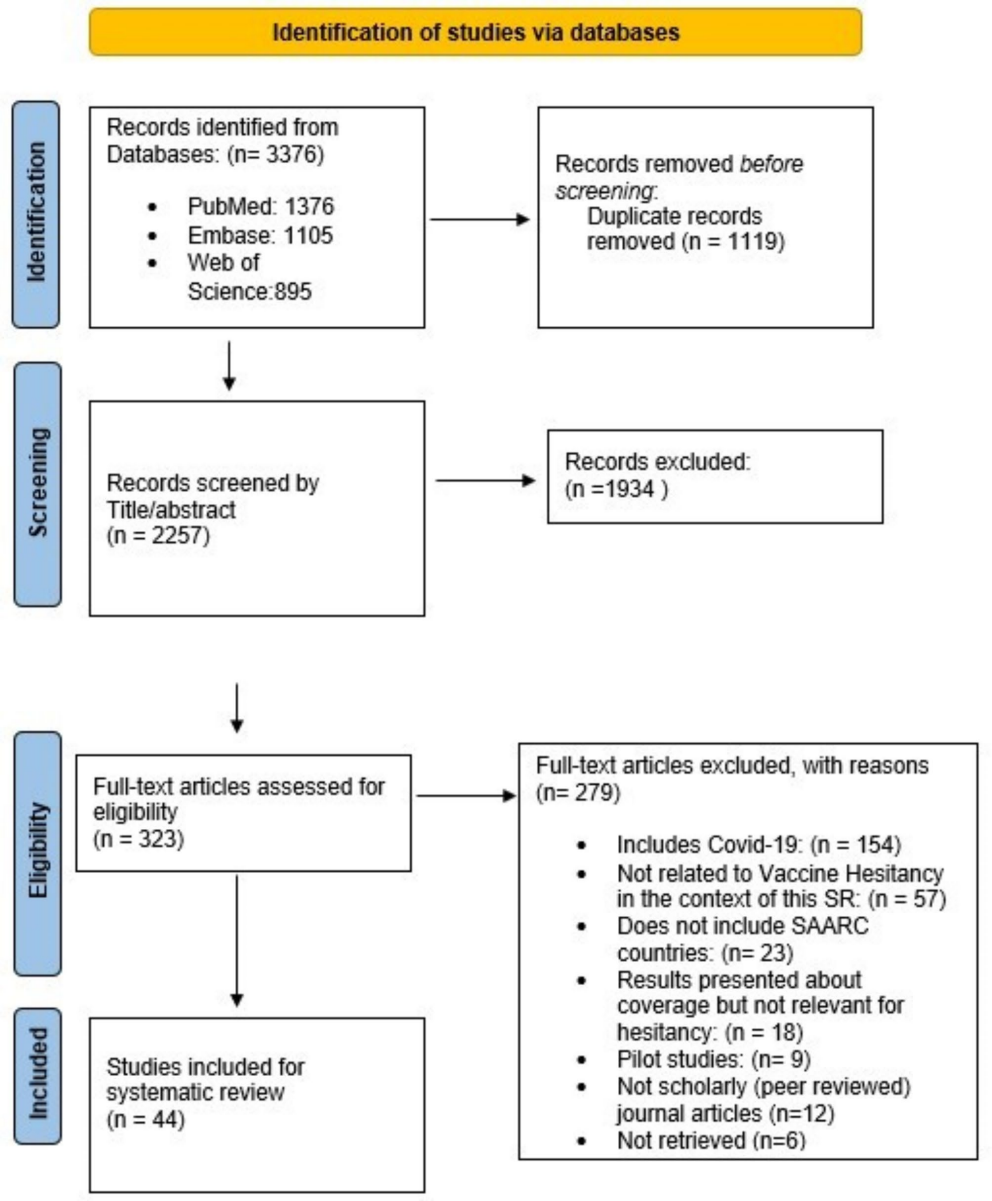
PRISMA flow diagram

Results of the search strategy are detailed below in Fig. 1 (Fig. 1). Out of 2257 papers after deduplication, 44 studies from five SAARC countries (India, Pakistan, Bangladesh, Nepal and Afghanistan) were included after full text screening (see table study characteristics SI 2) [35–78]. Results of the search strategy are detailed below.

While most of the included papers were from two countries, India (*n* = 22, 50%) and Pakistan (*n* = 16, 36. 63%), the study settings within the included countries were a mix of both rural and urban settings. Such diversity is also reflected in the variety of different study participants, ranging from caregivers, children, adolescents, medical personnel, low to high SES, different religions, local, national and international stakeholders, educated/uneducated and married/unmarried. Most of the studies focused on general childhood vaccines (*n* = 19), including Oral Polio Vaccine (OPV), BCG (tuberculosis vaccine), Pentavalent (Diphtheria, Pertussis, Tetanus, Hepatitis B, Haemophilus influenza type b (Hib)), PCV (Pneumococcal Conjugate) and Measles. Furthermore, studies individually addressed vaccines against Human Papillomavirus (HPV) (*n* = 6), OPV (*n* = 8), Influenza (*n* = 4), Measles-Rubella (*n* = 2), Pertussis (*n* = 1), Antenatal Influenza (*n* = 1), Hepatitis B (*n* = 1), Rotavirus (*n* = 1), Malaria (*n* = 1), RSV and “adult vaccines” (Zoster, Pneumococcal, HPV, Influenza, Varicella, Tetanus) (*n* = 1).

### Analysis of determinants of vaccine hesitancy

The following results first focus on the overall documented vaccine hesitancy. Further findings are mapped onto the 5 C framework [28]. Finally, we present the inductive themes of “knowledge and awareness” and “reasons to vaccinate”.

### Vaccine hesitancy

Similar to the vaccination coverage, vaccine hesitancy (VH) differed greatly between countries, settings, participants and vaccines. Studies reported varying rates of vaccine hesitancy or refusal for children’s vaccinations, with rates in Pakistan ranging from 8 to 61.3% [54–56, 60], and in India from 1.3% to as high as 83% [36, 51, 58, 59, 68]. For Afghanistan, only vaccine hesitancy toward OPV was documented with 21% [67].

Generally, study respondents who were most hesitant resided in lower resource areas (including urban slums), were of lower social status, lived further away from health clinics, and were more often unemployed when compared to their more affluent counterparts in both Pakistan and India [35–37, 41, 47, 51, 54, 55, 58–60, 68, 70, 71]. However, two studies showed the opposite, with higher hesitancy being found among higher income groups in the same countries [42, 70]. Employed fathers were less likely to hesitate or refuse to vaccinate their child in studies from Pakistan, compared to those from India where employed mothers were more likely to be vaccine hesitant, although this was not significant [51, 59]. Regarding education, studies painted a mixed picture. While lower education was significantly associated with incomplete immunization in studies from India and Pakistan, it was not necessarily due to VH. According to the included studies, the odds of VH were equally high between educated and non-educated parents for children’s vaccines [45, 46, 53, 70].

Vaccine hesitancy was further found to be associated with age and gender. Some studies evidenced that vaccine hesitancy increased with higher parental age [44, 51, 53, 57, 68], or for children of second or higher birth order [38, 70]. Further, out of the three studies that have examined vaccine hesitancy in relation with gender of the child, two have found higher odds of vaccine hesitancy among parents of male children [45, 70]. One study did not find a significant effect [36].

Vaccine hesitancy appeared to further vary with family size, pregnancy, minority status and religion. Pregnant women reported to be willing to have both their children as themselves vaccinated, findings in Karachi, Pakistan showed percentages as high as 83 and 87 for children’s vaccines respectively [45, 54]. Three studies in India [47, 59, 70] found that participants from nuclear families were more likely to be vaccine hesitant than joint families (an extended family, typically consisting of three or more generations and their spouses, living together as a single household). In specific, Cherian et al. [59] found that participants belonging to nuclear families were 2.3 times more vaccine hesitant versus those belonging to joint families. One study from Karachi documented a decreased likeliness to accept the Influenza vaccine among Bengalis, compared to Urdu-speakers [54]. Two studies from India addressed vaccine hesitancy among minorities and showed that families belonging to scheduled castes or scheduled tribes were less likely to be vaccinated, having 3.48 times greater odds of vaccine hesitancy compared to “other” castes [46, 50]. Although findings varied across settings, it seemed that for the Pentavalent vaccine, Measles, HPV and Influenza, the hesitancy proved to be somewhat higher than for other vaccines [41, 49, 53, 57, 58, 66]. Remaining studies conducted in Nepal did not include data on the prevalence of VH.

### The five C’s

#### Confidence

Confidence was divided into four sub-themes: fear of side effects, lack of faith in vaccination, lack of trust in government and lack of trust in HCWs.

#### Fear of side effects

Most studies (57% of those included) listed fear of side effects, adverse events or contraindications as a factor contributing to vaccine hesitancy. This includes six studies from Pakistan [35, 37, 40, 56, 60, 69], fourteen from India [36, 38, 41, 44–47, 50, 51, 57, 65, 66, 68, 71] and one from Bangladesh [61] covering childhood vaccines, adult vaccines, HPV, and Influenza. For childhood vaccines in urban Pakistan, six studies [35, 37, 40, 56, 60, 69] displayed that between 11.2 and 80.9% of parents feared side effects, with the rates ranging between 14 and 29% for OPV.

In India, fear of side effects from childhood vaccines, HPV and Influenza was higher among participants from urban areas, including middle-class and slum communities (9.92-80.8%) compared to rural areas (1.1–48%) [36, 38, 41, 44–47, 50, 51, 57, 65, 66, 68, 71].

Additionally, one study from Bangladesh, interviewing Rohingyas from refugee camps stated that participants were concerned that side effects may result in death [61].

#### Lack of faith in vaccination effectiveness

Six studies from Pakistan [35, 39, 40, 61, 65, 69], ten from India [44, 45, 49, 50, 52, 53, 57, 58, 68, 70], one from Nepal [72] and one from Bangladesh [61] identified lack of faith in vaccination as a factor of vaccine hesitancy. For Pakistan, two studies found that 4–19% of pregnant participants believed the Influenza and Pertussis vaccine to be harmful to them, their unborn child, that it may weaken their immune system and that it is overall ineffective [54, 55]. In terms of rural and urban settings, 5.1-37.7% of caregivers from urban areas in Pakistan did not trust childhood vaccines, considering them to be harmful (especially OPV) [35, 40, 65, 69]. In India, studies displayed that between 45 and 80% of caregivers and students from urban and rural areas equally distrusted vaccines [44, 45, 49, 50, 52, 53, 57, 58, 68, 70].

Furthermore, HCWs and caregivers in Kerala voiced lack of trust due to concerns that vaccines were not kept in ice, limited faith in the allopathic system of medicine that recommends immunization, and past negative experiences that have diminished trust in vaccination [63]. Lastly, Rohingyas from refugee camps in Bangladesh voiced safety concerns regarding childhood vaccines [61], along with 2.8% of medical students from Nepal in the context of Hepatitis B vaccination [72].

#### Lack of trust in government

Lack of trust in government was identified as a contributing factor to VH in four studies from Pakistan [37, 48, 56, 69], one from India [65] and one from Afghanistan [67]. In Karachi, study participants mistrusting the government ranged from 6.7 to 31.4% across three studies [37, 56, 69]. More specifically, participants residing in Charsadda, an urban center in North-West Pakistan, were hesitant to vaccinate their child against Polio because they did not trust in a government that fails to “provide employment and living requirements but has endless resources for the Polio campaign” [48]. In India, lack of trust in the government has also been reported. Civil society stakeholders and governmental officials participating in a study conducted in rural India, stated that people believe “the government may have an underlying agenda for putting so much effort into vaccination, while neglecting other issues”. In addition, healthcare provided by the government is perceived to be of low quality [65]. Lastly, 33% of caregivers from low-performing districts in Afghanistan mentioned lack of trust in institutions, organizing the OPV campaign [67].

#### Lack of trust in HCWs

With four studies from Pakistan [40, 42, 48, 69], two from India [52, 59], one from Bangladesh [61], Nepal [72] and Afghanistan [67], the lack of trust in HCWs is an important factor of vaccine hesitancy. 26% of HCWs of the polio eradication campaign stated that communities in Khyber Pakhtunkhwa (KPK) do not trust in their work, along with that, people believe that the reason for the HCWs frequent visits is to cover their mistakes in previous visits [40, 42]. In similar fashion, community members thought that vaccinators put water in the Polio- vaccines [48].

HCWs and caregivers from Nepal, Pakistan, Afghanistan and Bangladesh addressed a lack of mutual trust, microaggressions from clients, power imbalances, poor and limited quality of care, along with overall distrust in OPVs among 6.7–54% of respondents [61, 64, 67, 69]. In India, 4.9% of caregivers [59] and 23.4% of medical students from New Delhi [52] mistrusted immunization due to previous bad experiences with vaccinators and distrust in pharmaceutical companies in the context of receiving adult vaccines.

#### Complacency

In terms of complacency impacting vaccine hesitancy, 22 studies revealed complacent attitudes of participants [35–38, 40–47, 49, 50, 53, 57, 60, 65, 69, 70, 72, 74]. While complacency is impacted by several factors, such as confidence, convenience/constraints, communication, context, and knowledge around vaccines, the following results cannot be limited to intentional complacent behavior only.

Some participants perceived vaccination as unnecessary and to not be at risk to catch a disease. 4-50.6% of parents from studies in urban areas of India and Pakistan stated that they find childhood vaccination unnecessary because it does not protect their child, along with that diseases were perceived to be not common anymore and alternative ways of disease prevention were found to be more suitable [37, 40, 42, 46, 60, 65, 69, 70]. Furthermore, participants from studies in southern and western Indian urban areas expressed that Influenza did not require a vaccination, due to several reasons, such as the mildness of disease (5.7%), never having observed a severe case, low perceived risk and the fact that destiny determined illness (55%) as well as not being part of the risk group (42%) [44, 49, 57, 71]. For low and middle-income urban and rural areas, people believed in other preventative measures like herbs, masks, and Tamiflu to be better than an influenza vaccination [44]. Parents and caregivers were also shown to view RSV as non- severe, 61.2% believed bronchiolitis to be non-severe in children [74].

Moreover, the risk perception of contracting HPV and Hepatitis B varied between different population groups. While participants from as study in New Delhi perceived cervical cancer to not be as “popular” as other cancers [43], only 1.9% of medical students from Mangalore perceived the risk of infection to be low [38], this can be contrasted to 56.9% parents from rural Mysore [53]. This finding is significant as it reveals a major gap in communication disease related risks to parents. Similarly, only 0.6–3.3% of medical students from urban Nepal expressed hesitancy to vaccinate against Hepatitis B, with the minority who were hesitant not feeling at risk of developing the disease [72].

Lastly, postponement of childhood vaccination until another time was mentioned, by 8.7-9% of parents from a south Indian study, indicating different priorities [45]. The postponement of vaccination may indicate, amongst other reasons, a low prioritization of immunization.

#### Convenience/ constraints

21 studies [35, 37, 38, 40, 41, 43, 44, 48, 50, 52, 56–60, 65, 66, 68, 70, 72, 76] identified convenience and constraints as an influential factor of vaccination uptake. Here, four main themes became evident: accessibility, high expenses, lack of time and individual family constraints.

#### Accessibility

Long distance to place of immunization was mentioned by 13.8–59.4% of caregivers from studies in urban areas of Pakistan [35, 37, 60], along with 9.9-10.63% of medical students, doctors and families from studies in urban areas of India [36, 52, 59, 66]. While studies displayed similar numbers with regards to participants from rural India (9–11%), three studies specifically expressed transportation and storage issues with the delivery of Influenza, HPV, and childhood vaccines to rural areas [43, 44, 65]. Similarly, Khan et al. [48] stated that women in a rural area of north-west Pakistan were impossible to reach during the Polio-campaign. Riaz and colleagues [35] discovered that there is a strong association between children’s vaccination status and their distance from the health care center.

#### High expenses

Specifically, the distinction between vaccines included in the Routine Immunization (RI) schedule and those not covered under the Expanded Program on Immunization (EPI), such as the HPV vaccine in Mangalore, is crucial. While the NIP aims to eliminate direct costs for essential vaccines, many individuals remain hesitant or unable to access non-EPI vaccines due to financial constraints. In three studies from Pakistan [37, 56, 60], one study from Nepal [72] and seven studies from India [38, 41, 43, 44, 50, 57, 65], the high costs of adult and childhood vaccination posed a barrier to immunisation. Despite the fact the National Immunization Program (NIP) mostly covers the direct costs of vaccination in India, indirect costs present a common barrier [50, 65]. The majority of participants struggling with costs came from low SES [37, 38, 44, 56, 57, 60, 72]. Specifically for HPV, participants in a study of medical students in Mangalore indicated that the vaccine was too expensive at 1000 rupees [38]. While no difference between urban and rural areas was observed among studies from Pakistan and India, the number of participants complaining about high-costs was lower in studies in urban areas from India than from Pakistan [37, 41, 43, 56, 60].

#### Lack of time

Six studies from India and Pakistan identified lack of time and busy schedules as a reason for vaccine hesitancy, with 13-59.4% of participants from Pakistan [35, 40, 60]. and 2.09–10.63% from India [44, 52, 58].

#### Family situation and other reasons

Several different reasons related to family situation or other reasons were given for not accepting a vaccine. Erchick et al. [65] found that mothers from an Indian rural context hesitated to vaccinate their child as they had to fear domestic violence if their child cried after vaccination. Moreover, parents from slums in India mentioned that they forgot the date (7.5%), the mother was pregnant/ sick (10.6%), being away from home at the time of vaccination (6.8%) and discouragement by the family (6.2%). Some reported a miscellaneous family issue prevented parents to visit a local health center (8.9%) [70].

#### Communication

26 studies [35, 37–40, 42, 43, 45, 48, 49, 51, 52, 56, 57, 59, 61–69, 71, 75] displayed the effect of conspiracy theories/ rumors, miscommunication and external as well as internal influences through social media, religious leaders, and families on vaccine hesitancy. While religious conspiracies, and beliefs in underlying agendas of the government were described across all countries, they were particularly common in studies from Pakistan regarding OPV.

#### Conspiracy theories and rumors

In relation to conspiracy theories and rumors, six studies from Pakistan [39, 40, 42, 62, 69, 73] provided insight into common beliefs of participants surrounding OPV, HPV and childhood vaccination. For instance, 26.19–71.43% of urban study participants linked OPV to birth control or religious unacceptability, suggesting that the vaccine will reduce male population by causing sterility and that OPV is contaminated by pork [40, 42, 62, 69, 73]. This is similar to low-coverage districts in Afghanistan, where one paper found 31–53% of caregivers believed that OPV gives children polio, that it is not halal, or that OPV can give a child HIV/AIDS [67]. In Bangladesh, Ali et al. [61] found that 49% of unmarried adolescent girls hesitated to take the HPV vaccine due to negative rumors in the community.

Moreover, four studies from India [38, 45, 65, 66] showed that participants hesitated to receive vaccination due to various rumors or conspiracies. 2.6% of female students from Mangalore perceived HPV to be a disease of the low SES population [38], 9-53.3% of parents from rural areas heard rumors about adverse side effects from the Measles-Rubella vaccine and feared their child may die and develop complications [45, 66].

Although vaccination was understood as an important intervention to prevent childhood diseases, participants reported numerous barriers to vaccination. Strengthening vaccine demand and acceptance among displaced Rohingyas was suggested to be enhanced by improving vaccination delivery practices and engaging trusted leaders to address religious and cultural barriers using community-based channels [61].

#### Miscommunication

Like conspiracy theories and rumors, miscommunication enhances vaccine hesitancy. Based on the selected literature, lines between the two themes are easily blurred. A study from Pakistan found that 3.7–9.2% of parents from urban Pakistan received misinformation about contraindications from childhood vaccines, reported insufficient information about the venue and time of vaccination services and, wrongfully assumed to receive home-based services [35]. Two studies from India revealed that caregivers from urban India received negative information regarding childhood vaccines and that someone told them that their child suffered from bad reactions and the vaccine was not safe [68]. Women confused HPV with HIV because it was not explained at schools, and participants further believed that vaccination would encourage sexual behavior [43].

Four studies from India [49, 52, 57, 71] found miscommunication to be a factor in vaccine hesitancy, some university students expressed their fear of becoming an experimental animal [49], some parents explained that no one else was getting vaccinated for influenza [57], and women expressed hesitancy because doctors failed to recommend the antenatal influenza vaccine to them routinely [71]. Additionally, 7.3–53.6% of students, doctors and families from New Delhi revealed that HCWs did not recommend adult vaccines [49, 52], similar to participants from Bangladesh [61]. Lastly, Paul et al. [64] displayed that students from Nepal showed vaccine hesitancy due to lingering misconceptions and miscommunication.

#### Internal and external influence

When examining the impact of communication on vaccine hesitancy, it becomes essential to ask about the parties involved in the process of spreading (mis)information, conspiracies and rumours. Thereby, 11.9–78.6% of participants across India, Pakistan, Nepal and Afghanistan mentioned being influenced by religious or community leaders, neighbours, doctors, family members and older generations, like mothers- in law, advising against childhood vaccination [37, 42, 59, 62–68]. Furthermore, parents expressed negative social media videos as a main factor contributing to their hesitancy [59, 69].

#### Context

The overarching context of the involved countries impacting vaccine uptake can be divided into logistics around immunisation, views towards the government, religion, societal aspects, culture, and family traditions. While all Cs are potentially influenced by and entangled with *context*, this section lists the main findings.

#### Immunisation logistics and views towards the government

Fifteen studies [35, 37, 40, 44, 48, 50, 56, 58, 59, 64–66, 68, 72, 73] found immunisation logistics and views toward the government to influence vaccine hesitancy. It was found that caregivers from urban Pakistan and participants from urban India complained about absence of vaccinator, non-availability of vaccines, reduced clinic time, lack of immunization services for Polio, non-availability of healthcare-centers, overburdened HCWs, long waiting times and lack of trust in government [35, 37, 40, 50, 56, 58, 59, 65, 66, 68]. People with Pashtun ethnicity who already felt discriminated against, were suspicious of the state’s investment behind the Polio Eradication Program. Furthermore, vaccinators were accompanied by police or the mayor and people who were unwilling to vaccinate might have faced police force, which provoked disapproval [48]. On top of that, studies from India found that there is insufficient indication of vaccine priority from the government, along with lack of support from political leaders and lack of trust in the government believing that “they may have an underlying agenda for putting so much effort into vaccination while neglecting other issues” [44, 65, 66]. In Nepal medical students criticized the government for not providing a vaccination program for Hepatitis B [72].

Caregivers from Bangladesh voiced a lack of support from the Palika (rural municipality) and described the health facility environment to be “uncomfortable” in Bangladesh [61]. Next to that, 35% of HCWs faced security issues, as they commonly received threats during the polio eradication campaign in KPK [40]. Lastly, two studies from Pakistan and India respectively, described migration as a barrier to vaccination uptake [56, 65].

#### Religion

With regards to OPV uptake, HCWs from studies in north-west Pakistan, stated religious beliefs to impact vaccine hesitancy, because people were concerned that the vaccine was not halal and because the imam forbid polio vaccination [40, 42, 48]. Similar beliefs were found to be held regarding the conjugate typhoid vaccine Pakistan [75, 76]. Similarly, four studies from India [44, 52, 63, 66] displayed religious belief and the “faith in God to protect” to cause vaccine hesitancy. Certain Muslim or Christian study participants did not believe in being vaccinated because as one caregiver from Kerala states: “Allah is the one who gives disease to the child and if the child dies, it’s Allah’s destiny, why should we try to change that destiny by using vaccines?” [63]. In Bangladesh, participants from refugee camps explained that due to the Islamic principle of purdah, adolescent girls have to cover their bodies and cannot be seen by men outside of their family, therefore they are not allowed to go to public vaccination sites because vaccinators are mostly male [61].

#### Society and culture

Community members from urban Pakistan explained that OPV does not conform with the conservative Pashtun culture [48]. Four studies from India [38, 41, 43, 50] displayed that 8-13.22% of participants hesitated to receive the HPV vaccine due to social, or sexual stigma because sexual health is a taboo topic [38, 41, 43]. Francis and colleagues [50] found that mothers in India decided against childhood vaccination for their child if it was female.

#### Knowledge and awareness

Although *communication* partly embodies participants’ knowledge and awareness on vaccination (information may overlap), the 5 C model does not address it specifically. Therefore, it will be included as an additional overarching theme, like context, impacting all five Cs. Hereby, one may differentiate between knowledge/awareness regarding the logistics of vaccine uptake and general knowledge gaps leading to specific misconceptions and wrongful assumptions. In terms of logistics, 3.7- 26.68% of parents from urban Pakistan assumed that vaccination would be home-based, had insufficient knowledge about venue and time of vaccination services, reported on lack of knowledge regarding immunization schedule and the vaccination center’s location [35, 56], compared to only 0.17-5% of participants from urban and rural India [38, 50, 58, 66, 68]. Regarding knowledge gaps and resulting misconceptions, 8.07–35.5% of participants from urban Pakistan reported on lack of knowledge amongst patients for Polio and HPV, with knowledge being comparatively low for Hepatitis A vaccine, Chicken pox, MMR, and the Rotarix vaccine [39, 55, 77]. Along with that, lack of awareness of the need to get vaccinated for HPV, Influenza and childhood vaccines was slightly higher in urban and rural areas of India, with 2–56% of participants [43, 44, 50, 51, 63, 65]. That is due to lack of targeted information, educational materials, and insufficient knowledge from HCWs [71]. Studies from Nepal reported on similar findings in the context of Hepatits B [72]. Caregivers from Pakistan stated that “government is lagging in the awareness part, and they would wish to know what vaccines consist of” [48].

As a result of lacking knowledge and awareness, misconceptions started to linger, as previously touched upon under *communication*. For instance, 6.5–69% of participants from India, Bangladesh and Pakistan perceived vaccination to be harmful to their health, were unaware of what the vaccine consisted of, believed immunity by illness to be better, feared it may cause cosmetic issues, did not know about the need for follow-up doses, thought that multiple doses were harmful and that newer vaccines were riskier than older ones [35, 39, 42, 46, 47, 60–62, 65, 66, 68]. For HPV specifically, between 17.7% and 29.7% of Indian parents and female adults assumed HPV vaccination to not be required because they were not sexually active, while some believed the proper age to vaccinate was 40–50 years old or after puberty, and that HPV was genetically predisposed [38, 41, 43]. For influenza, residents from urban Pune believed that vaccines were only relevant for children under 1.5 years of age [44].

#### Reasons to vaccinate

While the focus is to understand people’s reasoning behind vaccine hesitant behavior or refusal of vaccination, mentioned reasons in favor of immunisation may provide important insights for future interventions. Studies found that most parents from rural and urban India and Pakistan agreed that childhood vaccines were crucial for the health of others and their child’s health, as vaccines provide effective and beneficial protection. A majority came to this conclusion by learning more about the diseases, witnessing related illness or death, and trusting in the information provided. Although many stated that the main reason for immunizing their child was to get them admitted to schools and because most parents did it. Along with that many chose to vaccinate, due to the vaccine’s cost-effectiveness, because it prevented diseases that would cause financial hardship [36, 44, 51, 52, 54, 55, 57, 68, 71]. In terms of influences, 5.7–41.1% were motivated by schoolteachers and HCWs or trusted the family physician’s advice, family, friends, other pregnant women (in the case of antenatal influenza) and media [44, 45].

With regards to recommendations that would impact vaccine uptake, study participants in India would agree that the acceptability of the HPV vaccine would increase if the government included it in the NIP [41]. Additionally, caregivers from Nepal recommended to improve knowledge and awareness, policy and national priority for full immunization, satisfaction and pride, social norms, generational shift in attitudes and instinctive compliance with HCWs [64].

## Discussion

In this paper we set out to examine the main factors influencing vaccine hesitancy in SAARC countries explained by the 5 C model and the two emerging themes *knowledge/ awareness* and *Reasons to vaccinate*. In reference to the displayed results, the main factors contributing to vaccine hesitancy in this review are shaped by a variety of components. Most of the papers included referred to data from Pakistan and India, one study from Afghanistan, one from Bangladesh and three from Nepal were included.

Overall, people who were most hesitant generally resided more often in low resource areas, were of lower social status, were further away from health clinics, belonged to scheduled casts, were older, unemployed, and of lower SES [35–37, 41, 44, 47, 51, 53–55, 57–60, 68, 70, 71]. Embedded into *context* and *constraints*, narrow confidence in vaccines, misleading communication, information seeking within close social networks, and lack of knowledge predominately constructed VH and refusal among involved participants. Along with that, VH mostly appeared to be somewhat disease specific, with HPV-VH being shaped by sexual and social stigma, Polio by contextual mistrust and conspiracies, and Influenza by complacency and lack of knowledge, while no discrete pattern could be found for childhood and adult vaccines generally. Moreover, no strong variation between SAARC countries was found indicating VH may not be entirely country-specific. Instead, there is variation within the countries between rural and urban areas, as well as high and low SES, although findings appear to be rather inconclusive in both India and Pakistan.

When compared to a systematic review by Dubé et al. [79] studying underlying factors of childhood vaccine hesitancy in high income countries (HICs), a few similarities as well as differences to this review’s findings of SAARCs becomes evident. While confidence and complacency regarding the fear of side effects, perceptions of vaccine necessity, mistrust in HCWs and institutions mostly appear similarly to this study’s findings, differences can be observed for *convenience/constraints*, *communication*,* context and knowledge*. While lack of knowledge and awareness posed as one of the main factors impacting VH in SAARC countries, participants’ knowledge in HICs was high and did not impact VH. Along with that, people in SAARC countries sought information within their own close social networks, whereas people in HIC mainly seek their information on the internet or science-based searches [62]. Furthermore, many participants from HICs did not face barriers in accessing vaccination services, because of high coverage and the fact that most vaccines are provided free of cost. While social stigma against immunization prevents people from getting vaccinated in SAARC countries according to this review’s findings, social stigma and norms almost force people to immunize their child or themselves in Dubé et al. [80] study on HICs, to avoid social judgement.

Digital communication and AI could pose as solutions to the struggles of older campaigns, for instance, as they help to reach more people, are more cost-effective, help to monitor, plan and collect data, they harbor just as many uncontrollable dangers like emerging infodemics, misconceptions and conspiracy theories [79].

On another note, people with higher education were sometimes more likely to be vaccine hesitant when compared to people with lower education. That may be due to better paying jobs that in turn allow for better healthcare or better access to knowledge on different vaccine types. While this may lead to less VH, it may also leave people with more available options about preferred vaccine types [81]. Moreover, general lack of knowledge and awareness on vaccination dominantly appeared across all countries, not only for “lay” people, but even for people trained in healthcare, such as HCWs, largely impacting the high prevalence of VH.

With regards to the 5 C model underlying this study, this review provided further detail to previously described interpretations of the different Cs. From the analysis in the present study, two new overarching themes, *knowledge and awareness* and *reasons to vaccinate* emerged in addition to the initial interpretations of the 5Cs. In this manner, it is important to consider positive aspects of the model as well as factors in need of improvement. On a positive note, the model allows researchers to gain insight into the great diversity of reasons impacting VH. Although this represents the multifactorial entanglement of components shaping health, the blurry lines between all Cs make it difficult to clearly distinguish whether decisions were made intentionally on an individual level or if they were unintentionally formed by distal circumstances. With that, is unclear whether all Cs are placed on the same level. Following the interpretation of this review, *context*, including religion and governmental decision-making, is placed on a higher level than *constraints/convenience* and the other Cs. Therefore, the question arises whether there is a hierarchy present within the model that is worthwhile considering going forward. Overall, the present study has shown that it can sometimes be difficult to interpret the findings according to the WHO SAGE definitions and previous studies. For that reason, it is worthwhile to consider that even though the 5Cs are interlinked, each C needs further detailing in order to clearly differentiate between them, when applying the extended version of the model that is based on this review’s findings. For instance, further detail is needed to explain the extent and discrete differences between *constraints/convenience* and *context*. Similarly, confidence and complacency seem to heavily overlap in studies considered in this review, depending on one’s interpretation. Along with that, one may argue about the role of complacency in the model, as it mainly entails blaming of participants, and mostly proved to be not suitable for the vaccines and circumstances discussed in this review. For instance, reasons like different priorities, disbelief in efficacy and the perception that vaccines are not necessary, represent complacent behavior according to the SAGE working group’s definition [15]. Based on that definition, previous studies placed more focus on individual calculation of risk versus collective responsibility, instead of emphasizing contextual and communicational influences [26, 82]. While some people perceive this to adequately represent complacency, this might entail blaming and belittling participants’ reasoning. Instead, one may ask whether people have different priorities due to financial or accessibility constraints and that the disbelief in vaccines is based on limited knowledge, awareness, lack of trust in institutions or miscommunication. Considering possible alternative explanations is therefore necessary when choosing to apply the 5 C model. Together with that, *complacency* might be more suitable to consider when analyzing data from HICs, especially regarding the COVID-19 pandemic, where a sort of “complacent movement” arose in alliance with conspiracy theories and misbeliefs. Based on the findings from this review, it becomes apparent that it is essential to move away from trying to blame participants for their complacent perceptions which may include calculation and their views on collective responsibility, but to take the context and ways of communication into account that shape those beliefs.

Moreover, the model does not include a “C” covering knowledge and awareness, which has proven to be one of the main factors influencing VH and refusal in this review. Considering its important role in the studies considered in this review, it was added to the model as a main component for which articles were analyzed. Given what we have seen with COVID-19, knowledge plays a prominent role everywhere, even though it may unfold in different ways. Therefore, researchers may consider including knowledge and awareness as an additional element for all countries. With that, future research should also explore the newly introduced 7 C and 8 C models, which enhance our understanding of vaccination readiness by refining factors, addressing overlaps, and providing a more detailed examination of complacency. In addition, these models incorporate time-sensitive criteria, including conspiracy theories and the psychosocial motivations for seeking vaccination solely for certification purposes [83].

Overall, applying the 5 C model as a theoretical model to this review has proven useful, as it provided guidance for analysis and further insights into practical implications and improvements. Despite its limitations, the model allows for the author to give their interpretation free rein, which may lead to bias but also encourages new innovative thinking.

Regarding recommendations for policy makers and global health professionals, it is crucial to tie in health and vaccination knowledge with community values, cultural perceptions and the overall context. It is particularly important to allow room for discussion and to engage all stakeholders and recipients, especially those that have a leading or authority position in the community. While policymakers should implement health and vaccine education at schools, so that children will use their knowledge to encourage a conversation at home, it is similarly important to engage parents and grandparents. Hence, as previously shown, internal influences from family members, oftentimes lead to hesitation or refusal of vaccines. In a similar vein, it is equally important to build mutual trust between people receiving vaccines and HCWs. The engagement of trusted civil society or religious leaders may help to achieve greater receptivity from people. Along with that, HCWs need to be trained to be able to tailor information on risks and benefits of vaccination (e.g., educated about religious beliefs) to the needs of the community. Together with that, the process of injecting vaccines may need to be re-evaluated through practice, so that patients experience as less pain as possible. Additionally, it is important for vaccination taking place in out-patient settings, to proceed in a private place shielded from other people’s reactions to create a safe space for recipients. Moreover, gender inequalities lead to a shortage in female HCWs in some areas which presents a barrier in female recipients for vaccination. Gender inequalities in such context are often perpetuated by wider policies or political decision making which admittedly is a difficult point to tackle. Preferably, future policies will problematize gender inequalities to improve the situation in this context. Finally, the social stigma surrounding specific vaccines needs to be diminished through country-fit awareness campaigns, indicating the government’s best interest in the health of all citizens. Along with evidence-based interventions that strengthen the role of HCWs and others with influence to increase equitable access to knowledge and services. Overall, trust in authorities needs to be rekindled, as it builds the foundation of vaccine uptake. This also entails combatting the high prevalence of conspiracy theories spreading through media platforms. In that manner, knowledge and awareness embody a key pillar that is shaped by open discussions between all generations, people of authority and HCWs based on the mutual willingness to learn, listen and respect one another with regards to individual fears, beliefs and circumstances. This may allow people to create a better understanding of certain views and misconceptions, enabling one another to adapt to a respectful and fruitful conversation around the importance of vaccines. While this conversation may take place between neighbors, friends, families, HCWs and patients or colleagues, it is crucial for the government to remember their influential role in this discussion. As this review has shown, it is particularly important to reappraise historical events that have caused citizens to mistrust the government’s intentions behind vaccination programs, which presents a long process that requires a great amount of resilience from all sides.

### Strengths and limitations

This systematic review has several strengths and limitations that should be considered when interpreting the presented results. Including 44 studies after careful screening and assessment allowed for a thorough analysis of the complex VH- spectrum. While previous studies mainly focused on solely one group of vaccines, the variety in different vaccines covered in this review allowed for a comprehensive overview and comparison between different drivers impacting VH. Furthermore, the inclusion of five SAARC countries and the great diversity in participants, socio-economic backgrounds and different regions within the countries allowed for results to be very nuanced and comprehensive. Such complexity needs to be considered when trying to understand VH in other settings.

However, this review contains a few limitations that need to be considered. As previously indicated the presented data is mainly attributed to selected areas within India and Pakistan, while fewer publications exist with data on Afghanistan, Bangladesh and Nepal. For the other SAARC countries, we were not able to find publications. However, this might be due to restricted full-text accessibility and language constraints. This raises the question to what extent data can be generalized to the SAARC region. The study also only included papers in the English or German language which will inevitably have an impact on the comprehensiveness and generalizability of the presented findings. Furthermore, there is the possibility of publication bias to impact the results of this review, with much research going unpublished due to publication bias but also due to conflict and social unrest disrupting ongoing research.

The present study focused on non-pandemic routine vaccinations. In conducting this systematic review of vaccine hesitancy, we deliberately excluded COVID-19 vaccines due to the unique and acute nature of the pandemic, which may have skewed perceptions of vaccine safety and efficacy. A different set of historical, socio-political and contextual factors shape vaccine hesitancy towards long-established vaccines and provide a better understanding of public sentiment. The rapid development and licensure of COVID-19 vaccines for emergency use introduced complexities that are not present with traditional vaccines, leading to fluctuating levels of trust and confidence. In addition, the evolving information about COVID-19 vaccines has contributed to an atmosphere of uncertainty that differs significantly from the stable knowledge surrounding other vaccines. Focusing on long-standing vaccines therefore allows for a clearer analysis of vaccine hesitancy based on more consistent public perceptions. Although considering what happened during the COVID-19 pandemic, the striking prominence of communication, especially misinformation and misbeliefs would have probably been even stronger had COVID-19 been included in the review. It is also possible that societal changes may have influenced participants perceptions during the pandemic, in that case results may be biased in both the direction of increased or decreased VH. Finally, the appliance of the theoretical 5 C model is prone to bias regarding data analysis, as previously indicated.

Notwithstanding these limitations, this review provides comprehensive data that could support public and global health professionals to understand key drivers of VH to develop suitable and sustainable solutions for VH.

Additionally, a methodological limitation of this study is the lack of a registered systematic review protocol in an open access repository such as PROSPERO.

## Conclusion

Altogether, a few key findings should be taken away from this systematic review examining contributing factors to vaccine hesitancy in SAARC countries. Firstly, the information studied in this review emerged mostly from the two countries, India and Pakistan, next to minimal data from Bangladesh, Nepal and Afghanistan. The remaining SAARC countries could not be covered, as we were unable to find suitable publications, however this might be due to restricted full text accessibility and language constraints. Secondly, VH appears to be contextual within SAARC countries rather than between countries, which has to do with varying settings, cultural, religious, and socio-economic context. Thirdly, underlying factors impacting vaccine hesitancy in SAARC countries are composed of narrow confidence in vaccines, misleading communication, and lack of knowledge embedded into overarching constraints and contexts. Especially, misinformation is a huge driver of VH that requires the development of interventions that are co-created by the people and communities they are applying to. Fourthly, the 5 C model needs to be further expanded in future research to paint an adequate picture tailored to specific contexts.

## Electronic supplementary material

Below is the link to the electronic supplementary material.

Supplementary Material 1

Supplementary Material 2

Supplementary Material 3

Supplementary Material 4

## Data Availability

All important data and results are included in this manuscript or supplementary files, all research the paper is based on is publicly available.

## Abbreviations

WHO: World Health Organization
SAGE: Strategic Advisory Group of Experts on Immunization
SAARC: The South Asian Association for Regional Cooperation
COIVD: 19–Coronavirus disease 2019
UNICEF: United Nations International Children’s Emergency Fund
DPT: Diphtheria Tetanus Pertussis
BCG: Bacillus Calmette–Guérin
HPV: Human Papilloma Virus
MMR: Measles Mumps Rubella
PRISMA: Preferred Reporting Items for Systematic reviews and Meta–Analyses
NICE: National Institute for Health and Care Excellence
OPV: Oral Polo Vaccine
PCV: Pneumococcal Conjugate Vaccine
VH: Vaccine Hesitancy
HCW: Health Care Worker
RSV: Respiratory Syncytial Virus
SES: Socioeconomic Status
KPK: Khyber Pakhtunkhwa
NIP: National Immunization Program
HIV/AIDS: Human immunodeficiency virus / acquired immunodeficiency syndrome
CIA: Central Intelligence Agency
US: United States
NGOs: Non–governmental Organizations
PROSPERO: International prospective register of systematic reviews
