# Supplementary material for: Factors influencing vaccine hesitancy toward non-covid vaccines in South Asia: a systematic review
Source: BMC Public Health. 2025 Apr 2;25:1246. doi: 10.1186/s12889-025-22462-4 (PMC11966902; doi:10.1186/s12889-025-22462-4)
Supplement: Supplementary file 1 — Supplementary Material 1 [file 12889_2025_22462_MOESM1_ESM.docx]

# **SI 1** Appendix 1 Search Strategy

|  | Date Final Search: 25.09.2024 | Results |
| --- | --- | --- |
| PubMed | ("asia, western"[MeSH Terms] OR ("India"[Title/Abstract] OR "Nepal"[Title/Abstract] OR "Bangladesh"[Title/Abstract] OR "Pakistan"[Title/Abstract] OR "Maldives"[Title/Abstract] OR "Sri Lanka"[Title/Abstract] OR "Bhutan"[Title/Abstract] OR "Afghanistan"[Title/Abstract] OR "south Asia"[Title/Abstract] OR "southern asia"[Title/Abstract] OR "British Indian Ocean Territory"[Title/Abstract]) AND ("vaccination refusal"[MeSH Terms] OR "anti-vaccination movement" [Title/Abstract] OR "vaccination"[MeSH Terms] OR "vaccines"[MeSH Terms] OR "immunization programs"[MeSH Terms] OR "preventative health service" [Title/Abstract] OR "vaccin*"[Title/Abstract] OR "immunisation"[Title/Abstract] OR "immunization"[Title/Abstract]) AND ("hesitanc*"[Title/Abstract] OR "refus*"[Title/Abstract] OR "accept*"[Title/Abstract] )) | 1376 |
| Embase | (exp South Asia/ or ('India' or 'Nepal' or 'Bangladesh' or 'Pakistan' or 'Maldives' or 'Sri Lanka' or 'Bhutan' or 'Afghanistan' or 'south Asia' or 'southern asia' or 'British Indian Ocean Territory').ti,ab,kf) and (exp vaccination refusal/ or exp anti-vaccination movement/ or ((exp vaccines/ or exp preventative health service/ or exp immunization/ or (vaccin* or immunization or immunization).ti,ab,kf ) and (hesitanc* or refus* or accept*).ti,ab,kf)) | 1105 |
| Web of Science | TS=(India OR Nepal OR Bangladesh OR Pakistan OR Maldives OR Sri Lanka OR Bhutan OR Afghanistan OR “south Asia” OR “southern Asia” OR “British Indian Ocean Territory” ) AND (TS=(anti-vaccination movement) OR (TS=(“preventative health service” OR “immunization” OR “immunisation” OR vaccine*) AND TS=(hesitanc* OR refus* OR “accept*”))) | 895 |
